# Berberine augments ATP-induced inflammasome activation in macrophages by enhancing AMPK signaling

**DOI:** 10.18632/oncotarget.13921

**Published:** 2016-12-12

**Authors:** Chen-Guang Li, Liang Yan, Yan-Yun Jing, Li-Hui Xu, Yi-Dan Liang, Hong-Xia Wei, Bo Hu, Hao Pan, Qing-Bing Zha, Dong-Yun Ouyang, Xian-Hui He

**Affiliations:** ^1^ Department of Immunobiology, College of Life Science and Technology, Jinan University, Guangzhou, China; ^2^ Department of Cell Biology, College of Life Science and Technology, Jinan University, Guangzhou, China; ^3^ Department of Nephrology, the First Affiliated Hospital of Jinan University, Guangzhou, China; ^4^ Department of Fetal Medicine, the First Affiliated Hospital of Jinan University, Guangzhou, China

**Keywords:** antibacterial infection, berberine, AMP-activated protein kinase (AMPK), inflammasome, macrophages, Immunology and Microbiology Section, Immune response, Immunity

## Abstract

The isoquinoline alkaloid berberine possesses many pharmacological activities including antibacterial infection. Although the direct bactericidal effect of berberine has been documented, its influence on the antibacterial functions of macrophages is largely unknown. As inflammasome activation in macrophages is important for the defense against bacterial infection, we aimed to investigate the influence of berberine on inflammasome activation in murine macrophages. Our results showed that berberine significantly increased ATP-induced inflammasome activation as reflected by enhanced pyroptosis as well as increased release of caspase-1p10 and mature interleukin-1β (IL-1β) in macrophages. Such effects of berberine could be suppressed by AMP-activated protein kinase (AMPK) inhibitor compound C or by knockdown of *AMPKα* expression, indicating the involvement of AMPK signaling in this process. In line with increased IL-1β release, the ability of macrophages to kill engulfed bacteria was also intensified by berberine. This was corroborated by the *in vivo* finding that the peritoneal live bacterial load was decreased by berberine treatment. Moreover, berberine administration significantly improved survival of bacterial infected mice, concomitant with increased IL-1β levels and elevated neutrophil recruitment in the peritoneal cavity. Collectively, these data suggested that berberine could enhance bacterial killing by augmenting inflammasome activation in macrophages through AMPK signaling.

## INTRODUCTION

Berberine is an isoquinoline alkaloid present in many medicinal herbs including *Rhizoma coptidis* and *Cortex phellodendri* as their major active ingredient [1]. Although possessing a variety of pharmacological effects including antidiabetic, anti-hyperlipidemic, antimicrobial, anti-inflammatory, and antioxidant activities, berberine has long been used mainly as an agent against gastroenteritis, dysentery, and abdominal pain [2]. Several reports have showed that its anti-gastroenteritic and anti-dysenteric effects are largely attributed to its direct antimicrobial effect on bacterial pathogens [3–7]. However, it is unknown whether berberine has potentiated the bacterial killing ability of the host's phagocytes including macrophages.

Macrophages are one type of innate immune cells distributed widely in different tissues, acting as the first line of defense against pathogenic infection. They not only kill bacteria by phagocytosis and present bacterial antigens to T and B lymphocytes, but also are responsible for damaged tissue repair [8]. When ingested by macrophages, bacterial pathogens become trapped in the phagosome, which is then fused with the lysosome to form the phagolysosome. Subsequently, hydrolytic enzymes and toxic peroxides kill the pathogens within the phagolysosome [9]. Engulfment of bacteria by macrophages may also cause the assembly and activation of large cytosolic multi-protein complexes known as inflammasomes [10, 11]. As a major consequence of inflammasome activation, the macrophages undergo pyroptosis while releasing inflammatory cytokines and danger signals, including interleukin-1β (IL-1β) and high mobility group box 1 (HMGB1). These molecules in turn recruit and activate other phagocytes such as neutrophils and monocytes [12], as well as enhancing their phagocytic and bacterial killing capacities [10]. Thus, induction of inflammasome activation is a robust mechanism for macrophages to fight against bacterial infection.

A variety of inflammasome pathways have been identified and one of the mostly investigated pathways is the nucleotide and oligomerization domain, leucine-rich repeat containing protein family, pyrin containing domain 3 (NLRP3) inflammasome [13]. It has been reported that the following two steps (signals) are required for the full activation of NLRP3 inflammasomes in murine macrophages [14]. Firstly, pattern recognition receptors (PRRs) expressed on macrophages recognize and bind to pathogen-associated molecular patterns (PAMPs) of bacteria, leading to the expression of critical components of the inflammasome, such as NLRP3 and pro-IL-1β. One well-known PAMP of Gram-negative bacteria is lipopolysaccharide (LPS). LPS stimulation of macrophages (i.e. LPS priming) induces a rapid expression of NLRP3 and pro-IL-1β, both of which are not expressed in unprimed macrophages [15]. Secondly, bacterial infection leads to the release of several danger signaling molecules termed damage-associated molecular patterns (DAMPs), which constitute an additional triggering signal for the assembly of NLRP3 inflammasomes. Following the assembly of NLRP3 inflammasomes, caspase-1 is recruited to the complex leading to its cleavage and activation, which subsequently catalyze the conversion of pro-interleukin-1β (pro-IL-1β) into mature IL-1β [14]. Activated caspase-1 can also lead to pyroptosis, which is required for the release of mature IL-1β [16, 17].

ATP is one well-known DAMP that can activate the NLRP3 inflammasome. ATP can be released by both the host innate immune cells and bacteria during microbial infection: upon PRR stimulation, monocytes/macrophages can release endogenous ATP into extracellular milieu [18], and macrophages can produce carbon monoxide (CO) to enhance ATP production by bacteria [19]. Extracellular ATP binding to its cell membrane receptor P2X_7_R activates NLRP3 inflammasomes and caspase-1, leading to the maturation and secretion of IL-1β [12], which in turn intensifies bacterial killing by the macrophages [19]. In support of this, recent studies revealed that caspase-1-deficient mice are more vulnerable to *Escherichia coli* infection [20], while bacterial pathogens can produce virulence factors that subvert inflammasome activation to benefit their persistence in the host [21–23]. Thus, ATP-induced inflammasome activation is critical for the clearance of bacterial pathogens [24].

In view of the pivotal role of ATP-induced inflammasome activation in the defense against bacterial infection and our preliminary data showing that berberine increased cell death in macrophages upon ATP treatment, we aimed to explore the effects of berberine on NLRP3 inflammasome activation in murine macrophages in response to ATP stimulation. We found that berberine treatment markedly enhanced ATP-induced inflammasome activation. Berberine also up-regulated AMP-activated protein kinase (AMPK) signaling, which was associated with inflammasome activation. Suppressing the AMPK activity significantly attenuated ATP-induced inflammasome activation. Importantly, berberine treatment intensified the bacterial killing ability of the macrophages *in vitro* and *in vivo*, thus increasing the survival of mice with bacterial infection. Our findings highlight the indirect antimicrobial effects of berberine against bacterial infection by promoting NLRP3 inflammasome activation and thus augmenting the functions of macrophages.

## RESULTS

### Berberine enhances ATP-induced inflammasome activation in murine J774A.1 macrophages

**Figure 1:**
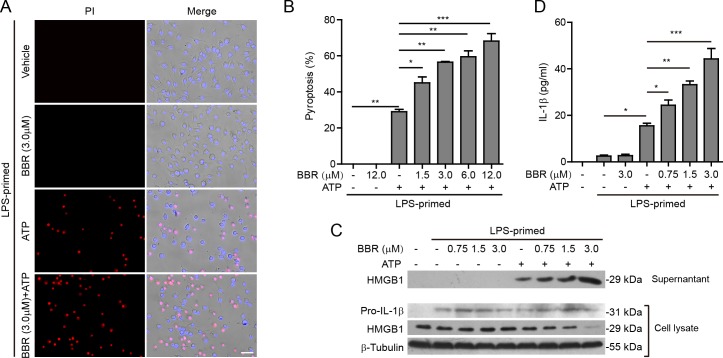
Berberine enhanced ATP-induced inflammasome activation in murine J774A.1 macrophages **A.** Cells were primed with LPS (500 ng/ml) for 4 h and then pre-treated with graded concentrations of berberine for 1 h, followed by incubation with ATP (3 mΜ) for 1 h in the absence of LPS. Cell death was assayed by propidium iodide (PI) (red; staining dead cells) and Hoechst 33342 staining (blue; staining all cells). The fluorescent images were captured by fluorescence microscopy, merged with bright-field images. One set of representative images of three independent experiments are shown. Scale bar, 50 μm. **B.** PI-positive cells in 5 randomly chosen fields each containing ~100 cells were quantified. Data are shown as mean ± SD (*n* = 5). **C.** Cells were treated as in **A.**. Western blotting was used to assess the expression levels of indicated proteins in the cell lysates and culture supernatants, respectively. β-Tubulin was used as a loading control for cell lysates. **D.** Quantification of soluble IL-1β levels in the culture supernatants by cytometric bead array (CBA) assay. Data are shown as mean ± SD (*n* = 3). *P < 0.05; **P < 0.01; ***P < 0.001; BBR, berberine.

As a common *in vitro* bacterial infection cellular model, J774A.1 cells are often first primed with LPS (first signal), and then stimulated with ATP (second signal) to activate NLRP3 inflammasome. Our preliminary experiments showed that berberine incubation for 48 h at doses lower than 20 μM had no significant cytotoxicity on J774A.1 cells (data not shown). Hence, the berberine doses were lower than 20 μM in the following experiments. As cells undergoing pyroptosis after NLRP3 inflammasome activation can be monitored by propidium iodide (PI) staining [25], we used this approach to assay ATP-induced pyroptosis. After LPS priming, ATP treatment could induce pyroptosis in ~30% of J774A.1 cells (Figure 1A and 1B). Notably, berberine treatment before ATP stimulation markedly increased the pyroptosis in a dose-dependent manner as compared with ATP (alone) group (Figure 1B). This was corroborated by increased release of HMGB1 into the culture supernatants accompanied by a decrease of HMGB1 levels in the cell lysates (Figure 1C). Without ATP triggering, however, berberine *per se* did not induce pyroptosis and HMGB1 release into the supernatants. Although pro-IL-1β expression was up-regulated after LPS priming, mature IL-1β (17 kDa) was undetectable in all the culture supernatants by western blotting. Therefore, we detected the mature IL-1β levels in the supernatants by using a bead-based immunoassay (CBA), and found that berberine dose-dependently increased IL-1β release (although at low levels) upon ATP stimulation (Figure 1D). Together, these results indicated that berberine could augment the NLRP3 inflammasome activation in murine J774A.1 macrophages upon ATP stimulation.

### Berberine enhances ATP-induced inflammasome activation in primary macrophages

**Figure 2:**
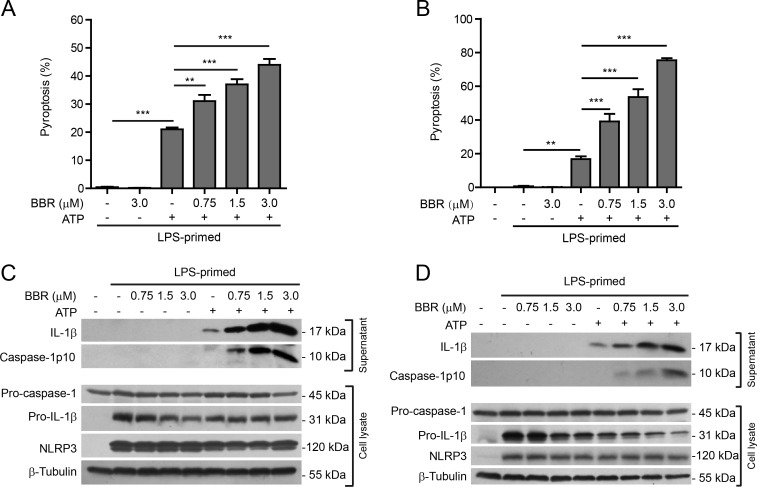
Berberine increased ATP-induced inflammasome activation in primary murine macrophages Thioglycollate-elicited peritoneal macrophages (TGPMs) and bone marrow-derived macrophages (BMDMs) were first primed with LPS (500 ng/ml) for 4 h and then pre-treated with indicated doses of berberine for 1 h, followed by incubation with ATP (2 mΜ) for 30 min without LPS. **A.** and **B.** Cell death in TGPMs **A.** and BMDMs **B.** was assayed by propidium iodide (PI) (for dead cells) and Hoechst 33342 (for all cells) staining and quantified by counting 5 randomly chosen fields each containing ~100 cells. Data are shown as mean ± SD (*n =* 5). **P < 0.01; ***P < 0.001. **C.** and **D.** Western blot analysis was used to assess the expression and secretion of caspase-1, IL-1β and other proteins in the cell lysates and culture supernatants in TGPMs **C.** and BMDMs **D.**, respectively. β-Tubulin was used as a loading control for cell lysates. BBR, berberine.

To confirm the results observed in J774A.1 cell line, we next sought to explore whether berberine had similar effects on NLRP3 inflammasome activation and pyroptosis in primary murine macrophages. Both thioglycollate (TG)-elicited peritoneal macrophages (TGPMs) and bone marrow-derived macrophages (BMDMs) were used as primary macrophage models. The former cells represent inflammatory macrophages while the latter are unstimulated ones. These cells were first primed with LPS, and then pre-treated with graded doses of berberine prior to ATP treatment. Similar to the results from J774A.1 cells, berberine dose-dependently enhanced ATP-induced pyroptosis in both TGPMs and BMDMs, but it alone did not cause any cell death (Figure 2A and 2B). Morphologically, berberine-enhanced cell death was similar to ATP-induced pyroptosis, with rapid membrane permeabilization and cellular swelling but no condensation of nuclei (Supplementary Figures S1 and S2).

In either TGPMs (Figure 2C) or BMDMs (Figure 2D), both pro-IL-1β and NLRP3 proteins were highly induced after LPS priming, whereas pro-caspase-1 was constitutively expressed regardless of LPS priming. Upon further stimulation with ATP, both mature IL-1β (17 kDa) and active caspase-1p10 were released into the culture supernatants, indicating inflammasome activation. Again, berberine treatment increased the release of these contents upon ATP stimulation in these cells (Figure 2C and 2D). Berberine alone caused a slight decrease in pro-IL-1β levels but no significant changes in pro-caspase-1 levels in the cell lysates; it could further decrease pro-IL-1β levels in ATP-treated BMDMs but not TGPMs. Without ATP treatment, however, both TGPMs and BMDMs did not display inflammasome activation (Figure 2C and 2D). Altogether, these results indicated that berberine enhanced ATP-induced NLRP3 inflammasome activation in a dose-dependent manner in both TGPMs and BMDMs, in line with the results from J774A.1 cells.

**Figure 3:**
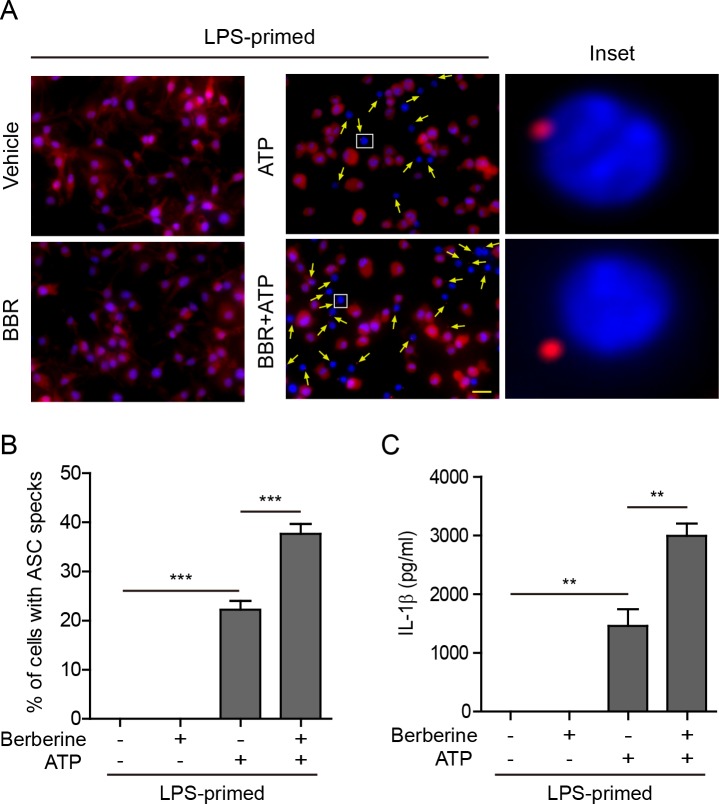
Berberine increased ATP-induced ASC speck formation and IL-1β release in bone marrow-derived macrophages (BMDMs) Mouse BMDMs were primed with LPS (500 ng/ml) for 4 h and then pre-treated with berberine (3 μM) for 1 h, followed by incubation with ATP (2 mΜ) for 30 min without LPS. **A.** Representative immunofluorescence images showing ASC sub-cellular distribution. Cells were stained with anti-ASC antibody (red) and Hoechst 33342 (blue). The images for ASC and nuclei were captured respectively and merged together. Arrows indicate ASC specks and the insets show the enlarged cells with an ASC speck. Scale bar, 20 μm. **B.** The percentages of cells containing ASC specks were calculated by the number of cells with ASC specks relative to the total number of cells from 5 random fields each containing ~50 cells. Data are shown as mean ± SD (*n =* 5). **C.** Quantification of soluble IL-1β levels in the culture supernatants by CBA assay. Data are shown as mean ± SD (*n* = 3). **P < 0.01; ***P < 0.001; BBR, berberine.

Two additional assays were performed in BMDMs, which further confirmed that berberine enhanced ATP-induced inflammasome activation. Firstly, immunofluorescence microscopy was used to analyze the percentages of cells containing ASC specks (a marker of inflammasome activation). ASC was distributed evenly in LPS and vehicle-treated cells, whereas ASC specks were formed in ~20% of the cells upon ATP treatment (Figure 3A and 3B). Pre-treatment with berberine before ATP stimulation increased the percentages of cells containing ASC specks to ~40%, corroborating enhanced activation of NLRP3 inflammasomes by berberine. Secondly, the CBA assay was used to measure the soluble IL-1β in the culture supernatants. The results showed that ATP treatment induced IL-1β release from LPS-primed BMDMs, while berberine treatment further increased the IL-1β level in the supernatant (Figure 3C), thus confirming the result of the western blot analysis of IL-1β (Figure 2D).

### Berberine increases AMPK signaling in macrophages upon ATP treatment

**Figure 4:**
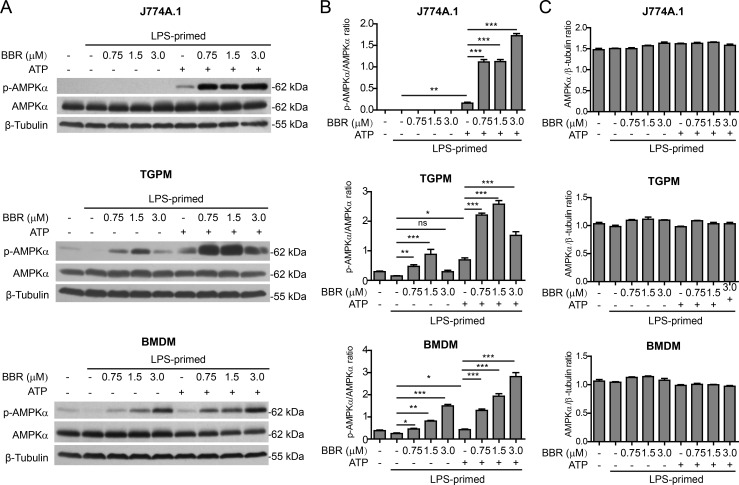
Berberine enhanced AMPK signaling in macrophages upon ATP treatment Mouse macrophage J774A.1 cells, thioglycollate-elicited peritoneal macrophages (TGPMs) and bone marrow-derived macrophages (BMDMs) were treated as described in Figure 1 and Figure 2, respectively. Western blotting was used to assess protein expression in the cell lysates **A.** β-Tubulin was recruited as a loading control. Quantifications of p-AMPKα relative to AMPKα are shown in **B.**, while quantifications of AMPKα relative to β-tubulin are shown in **C.**, respectively (*n =* 3). *P < 0.05; **P < 0.01; ***P < 0.001; ns, not significant; BBR, berberine.

As berberine has been reported to be an AMPK agonist [26, 27], we next explored whether AMPK activation by berberine contributed to potentiating ATP-induced inflammasome activation. AMPK activity can be evaluated by the phosphorylation at Thr172 of AMPKα, the kinase subunit of AMPK heterotrimeric complex [28]. Western blot analysis showed that the AMPK activity was suppressed by LPS in J774A.1 cells, TGPMs and BMDMs (Figure 4A and 4B). ATP treatment induced AMPK re-activation in all these LPS-primed macrophages. As expected, berberine treatment activated AMPK in TGPMs and BMDMs, despite the presence of LPS. Notably, ATP-induced AMPK activation was greatly enhanced by berberine pre-treatment in all these cells (Figure 4A and 4B), whereas the levels of total AMPKα protein were unaffected by these treatments (Figure 4A and 4C). Together with the above-mentioned data, these results suggested that berberine-induced AMPK signaling was associated with the augmentation of ATP-induced inflammasome activation.

### Blocking AMPK signaling attenuates berberine-mediated augmentation of ATP-induced inflammasome activation

**Figure 5:**
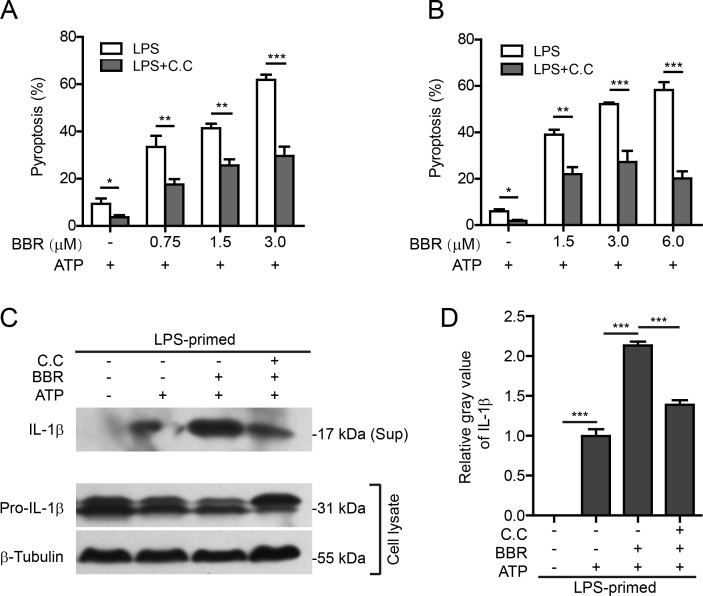
AMPK signaling blockade attenuated berberine-mediated enhancement of ATP-induced inflammasome activation **A.** After primed with LPS (500 ng/ml) for 4 h, J774A.1 cells were pre-treated with AMPK inhibitor compound C (C.C) (20 μΜ) for 1 h before incubation with indicated concentrations of berberine for 1 h followed by co-treatment with ATP (3 mΜ) for 1 h. Cell death was assayed by propidium iodide staining. Data are shown as mean ± SD (*n* = 5). **B.** TG-elicited peritoneal macrophages were sequentially treated with LPS (500 ng/ml for 4h), C.C (20 μΜ for 1 h), berberine (indicated doses, for 1 h) and ATP (2 mM for 30 min). Cell death was assayed by propidium iodide staining. **C.** TG-elicited peritoneal macrophages were treated as **B.** except that berberine concentration was 3 μM. Western blotting was used to evaluate the expression and secretion of IL-1β in the cell lysates and culture supernatants (Sup), respectively. β-Tubulin was used as a loading control for cell lysates. **D.** Histograms showing the relative gray values of IL-1β in **C.** (*n* = 3). The gray value of IL-1β band in ATP group was set as 1.0. The gray values of the other groups were calculated relative to the ATP group. *P < 0.05; **P < 0.01; ***P < 0.001; BBR, berberine.

To further confirm that berberine promoted inflammasome activation by inducing AMPK signaling, specific AMPK inhibitor compound C (C.C) or small-interfering RNA (siRNA) were used to suppress the AMPK activity. Firstly, J774A.1 macrophages and TGPMs were primed with LPS, and then treated with C.C for 1 h. After incubation with berberine for 1 h, the cells were treated with ATP to induce NLRP3 inflammasome activation and pyroptosis. The results showed that both ATP-induced pyroptosis and berberine-mediated augmentation of ATP-induced pyroptosis were significantly attenuated by C.C pre-treatment (Figure 5A and Supplementary Figure S3; Figure 5B and Supplementary Figure S4). In line with the pyroptosis data, berberine-promoted release of mature IL-1β (17 kDa) into the culture supernatants of TGPMs was also markedly decreased by C.C pre-treatment (Figure 5C and 5D). The pro-IL-1β level appeared to be reduced by ATP and berberine co-treatment but was recovered by C.C treatment (Figure 5C). These results indicated that berberine-mediated enhancement of inflammasome activation was reversed by pharmacological inhibition of AMPK activity.

**Figure 6:**
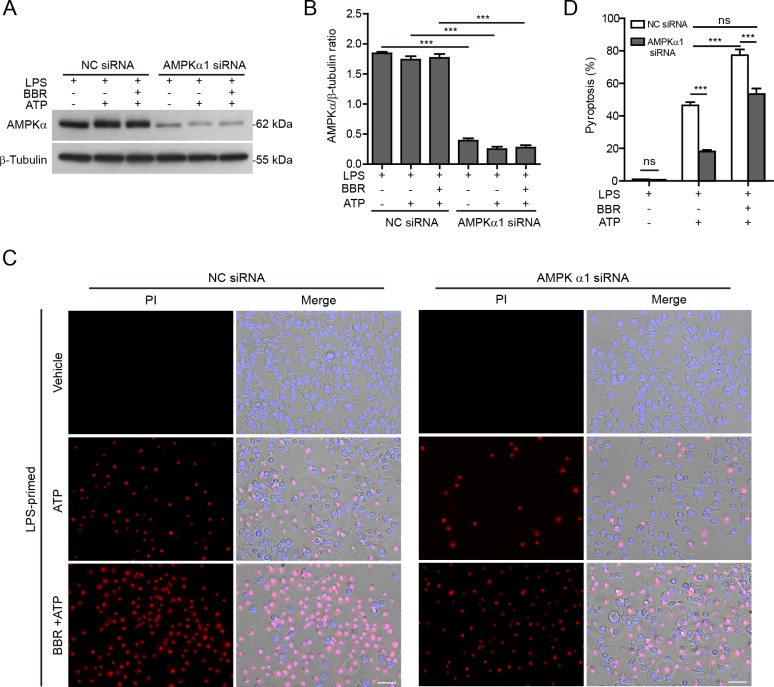
AMPKα knockdown attenuated ATP-induced inflammasome activation in macrophages J774A.1 cells were transfected with negative control (NC) siRNA or *AMPKα1*-specific siRNA for 72 h. Then the cells were primed with LPS (500 ng/ml) for 4 h, and pre-treated with berberine (3 μM) for 1 h followed by co-treatment with ATP (3 mΜ) for 1 h without LPS. **A.** Cell lysates was analyzed by western blotting. β-Tubulin was recruited as a loading control. **B.** Histograms show the quantification of AMPKα levels relative to β-tubulin in **A.** (*n* = 3). **C.** Cell death was assayed by propidium iodide (PI) (red) and Hoechst 33342 (blue) staining, and fluorescent images were captured by fluorescence microscopy, merged with bright-field images. One representative set of images of three independently performed experiments are shown. Scale bars, 50 μm. **D.** PI-positive cells in 5 randomly chosen fields each containing ~100 cells were quantified. Data are shown as mean ± SD (*n* = 5). ***P < 0.001; ns, not significant; BBR, berberine.

Secondly, the expression of AMPKα (the kinase subunit of AMPK) in J774A.1 cells was knocked down by siRNA. Western blotting showed that the expression of AMPKα protein was reduced approximately 70% after knockdown (Figure 6A and 6B). Consistent with the pharmacological observation, AMPKα knockdown also attenuated berberine's effect on promoting the inflammasome activation (as reflected by pyroptosis) in response to ATP stimulation (Figure 6C and 6D). Notably, although berberine was present, the pyroptosis rate induced by ATP treatment in the cells with AMPKα knockdown was comparable to that in the cells treated with negative control (NC)-siRNA in the absence of berberine. As ATP treatment induced AMPK activation, AMPKα knockdown also reduced ATP-induced pyroptosis in the absence of berberine (Figure 6C and 6D). Altogether, these results indicated that AMPK signaling at least partly contributed to berberine-mediated augmentation of ATP-induced inflammasome activation.

### Berberine administration enhances bacterial killing and prolongs mouse survival against bacterial infection

**Figure 7:**
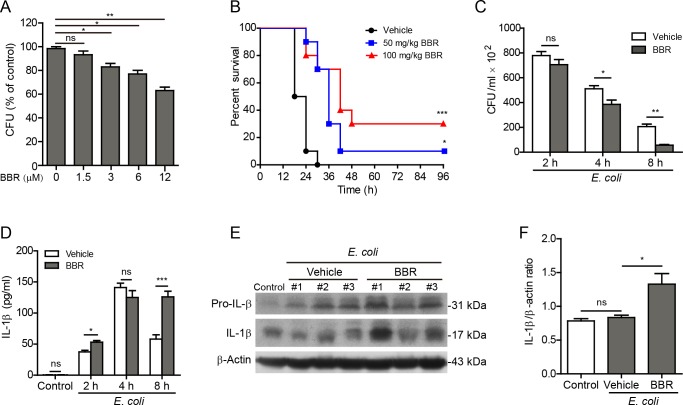
Berberine enhanced bacterial killing and prolonged mouse survival during bacterial infection **A.** J774A.1 cells infected with *E. coli* for 2 h, followed by incubation with various concentrations of berberine for 4 h. Then the engulfed bacteria were freed from the cells and the colony-forming units (CFUs) were measured by Luria Broth (LB) agar culture. **B.**-**F.** C57BL/6 mice were administered (i.g.) with berberine (50 and 100 mg/kg body weight **B.** or 100 mg/kg body weight **C.**-**F.**) or vehicle (2% Tween-80 in PBS) once a day for 3 consecutive days prior to infection (i.p.) with viable *E. coli* (2×10^9^ CFU/mouse). **B.** Mouse survival was monitored every 6 h for 4 days. Kaplan-Meier survival curves were used to analyze the data (10 mice per group). The significance was evaluated by the log-rank (Mantel-Cox) test. **C.** Viable bacterial counts (CFUs) in the peritoneal cavity of *E. coli*-infected mice were measured by using LB agar culture. **D.** The levels of IL-1β in the peritoneal exudates from the mice were measured by CBA assay (*n* = 5). **E.** Western blotting of colonic tissue lysates (8 h post bacterial infection). β-Actin was used as a loading control. **F.** Quantification of IL-1β levels relative to β-actin in **E.** (*n* = 3). *P < 0.05; **P < 0.01; ***P < 0.001; ns, not significant; BBR, berberine.

We next examined the effect of berberine on bacterial killing by macrophages. Equal amounts of *E. coli* were added to and phagocytosed by macrophages and the colony-forming units (CFUs) of the engulfed bacteria within the macrophages after berberine treatment were analyzed. As shown in Figure 7A, berberine significantly increased bacterial killing by macrophages in a dose-dependent manner.

Finally, we explored whether berberine augmented inflammasome activation and IL-1β secretion *in vivo* in the context of bacterial infection in the peritoneal cavity of mice, which is a commonly used bacterial infection model. Mice were administered intragastrically with berberine (50 or 100 mg/kg body weight) or vehicle once a day for 3 consecutive days followed by intraperitoneally injection with a lethal dose of viable *E. coli* (2×10^9^ CFU/mouse). Such a dose of bacteria killed control mice within 30 h, whereas berberine pre-treatment significantly prolonged their survival (Figure 7B). In low-dose (50 mg/kg) and high-dose (100 mg/kg) berberine groups, 10% and 30% of mice survived the period of observation (96 h), respectively. This prolonged survival of mice might be due to enhanced bacterial killing and clearance by the peritoneal macrophages and/or neutrophils because berberine significantly decreased the bacterial load (CFUs) in the peritoneal cavity when compared with vehicle (Figure 7C). In line with enhanced bacterial killing, the secreted mature IL-1β was markedly elevated at the time points of 2 h and 8 h in the peritoneal lavage fluids of berberine-administered mice as compared with vehicle-treated ones, albeit there was no significant difference at the time point of 4 h (Figure 7D); this suggested that berberine augmented inflammasome activation *in vivo* upon bacterial infection. Consistent with this, the levels of mature IL-1β (17 kDa) in the colonic tissues were also significantly increased in berberine-treated mice compared with vehicle (Figure 7E and 7F). These results suggested that berberine administration prolonged mouse survival by enhancing inflammasome activation and bacterial killing in the infected sites.

**Figure 8:**
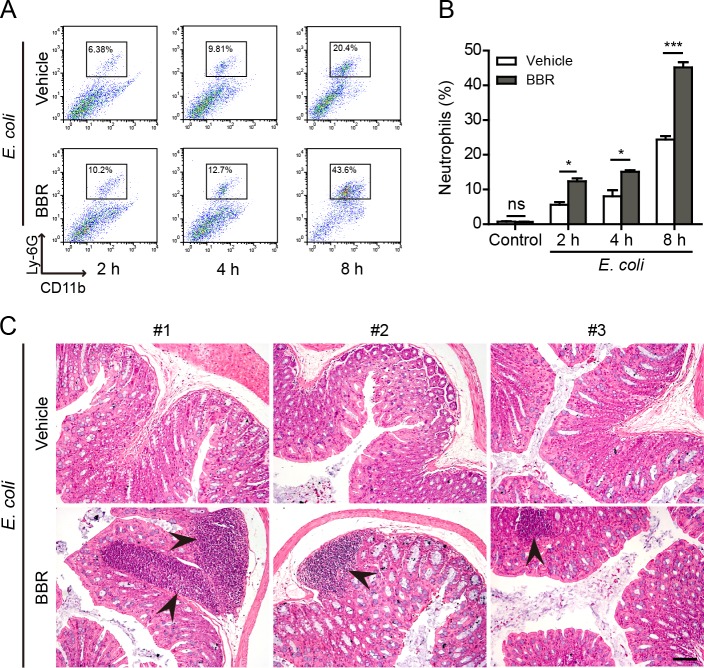
Berberine increased neutrophil recruitment in the peritoneal cavity and the colon C57BL/6 mice were administered (i.g.) with berberine (100 mg/kg body weight) or vehicle (2% Tween-80 in PBS) once a day for 3 consecutive days prior to injection (i.p.) with viable *E. coli* (2×10^9^ CFU/mouse). After indicated time periods, cells in the peritoneal cavity were harvested by 1.5 ml PBS and double stained with FITC-CD11b and PerCP-Gr-1 antibodies for flow cytometric analysis, and the colon was collected and fixed in 4% neutral formaldehyde for histological analysis. **A.** Representative flow cytometric dot-plots of three independent experiments. **B.** Quantification of CD11b^+^ Gr-1^+^ cells (neutrophils) within the gate in **A.** Data are expressed as mean ± SD (*n* = 3). *P < 0.05; ***P < 0.001; ns, not significant. **C.** Hematoxylin and eosin (H&E) staining of the colon sections 8 h after infection (*n =* 3). Representative images from each mouse are shown. Arrows indicate infiltrated inflammatory cells in the colon tissues. Scale bar, 100 μm; BBR, berberine.

As IL-1β release was increased by berberine treatment in the mouse model, we examined whether berberine increased neutrophil recruitment in the peritoneal cavity upon bacterial infection. Indeed, flow cytometric analysis revealed that the percentages of neutrophils (CD11b^+^Gr-1^+^) in the peritoneal cavity of mice were markedly increased by berberine administration as compared with vehicle (Figure 8A and 8B). Consistent with the increase of mature IL-1β levels in the colonic tissues of berberine-treated and bacterial-infected mice, inflammatory foci with a large number of inflammatory cells could be observed beneath the colon wall of these mice but not those treated with vehicle (Figure 8C). These infiltrated cells in the foci were mainly neutrophils as judged by their polymorphonuclear feature (Supplementary Figure S5). However, berberine did not increase the infiltration of inflammatory cells in the liver (Supplementary Figure S6). These results suggested that berberine might promote the recruitment of inflammatory cells by augmenting inflammasome activation in the infected sites.

## DISCUSSION

Berberine possesses many pharmacological activities, including anti-gastroenteritic and anti-dysenteric effects, but the underlying mechanism(s) is only partly understood. In this study, we provided a new explanation for its antimicrobial activity other than its direct bactericidal effect as previously reported [2, 4, 7, 29]. Our data showed that berberine significantly augmented the NLRP3 inflammasome activation in response to ATP triggering, resulting in increased pyroptosis and release of mature IL-1β, in murine macrophages. Consistent with these *in vitro* results, oral administration of berberine to mice markedly elevated the levels of soluble IL-1β in the lavage fluids of their peritoneal cavities upon bacterial infection. Moreover, treatment with berberine enhanced bacterial killing within macrophages. Importantly, berberine administration not only reduced the bacterial load in the peritoneal cavity but also increased the survival of bacterial infected mice. These data indicated that berberine could indirectly exhibit its antimicrobial activity by enhancing the functions of macrophages upon microbial infection.

Inflammasome activation is a critical defense mechanism of the innate immune system against microbial infection [12]. One outcome of inflammasome activation is maturation and release of IL-1β, as well as other inflammatory cytokines [12]. These cytokines not only promote the functions of phagocytes by increasing their phagocytic and bacterial killing abilities [18, 30, 31], but also are necessary for the recruitment of other immune cells including neutrophils and monocytes/macrophages [32, 33]. Consequently, induction of inflammasome activation and IL-1β release can intensify the host defense against bacterial infection in murine models [10, 19, 33, 34]. Another study revealed that bacterial infection induces carbon monoxide (CO) production in macrophages, which in turn promotes their bacterial killing and phagocytosis abilities both *in vitro* and *in vivo* through increasing the activation of NLRP3 inflammasome and the secretion of active IL-1β [19]. Consistent with these studies, we observed that berberine treatment increased IL-1β production (suggestive of inflammasome activation) upon bacterial infection *in vivo* or upon LPS+ATP stimulation *in vitro* which resembles the circumstance of bacterial infection. The bacterial killing ability of macrophages was significantly enhanced by berberine treatment. More neutrophils were recruited into the peritoneal cavity and colonic inflammatory foci of berberine-administered mice upon bacterial infection, which increased bacterial clearance. The survival of these mice was significantly improved by berberine. Therefore, our data demonstrated that berberine could enhance bacterial killing by augmentation of inflammasome activation in macrophages during bacterial infection.

Recent studies have indicated that the release of mature IL-1β, is dependent on pyroptosis [35], another consequence of inflammasome activation. In line with this, we found in this study that the extracellular levels of IL-1β consistently correlated with the rate of pyroptosis, and berberine treatment increased both IL-1β release and pyroptosis upon ATP stimulation. Other products of the inflammasome pathway might also be released dependently on pyroptosis, such as HMGB1 (having been shown to be released by macrophages in this study) and gasdermin D N-terminal fragments (GSDMD-N). HMGB1 is an important danger signal acting like a cytokine to stimulating immune cells [36]. And GSDMD-N is produced by active caspase-1 or caspase-11, with pore-forming activity to kill bacterial pathogens directly [16, 17]. Although it remains to be discovered whether such direct bacterial killing plays a role in controlling *in vivo* infection, we presumed that the release of GSDMD-N should be enhanced by berberine due to increased pyroptosis during bacterial infection. Yet more sophisticated approaches are needed to observe the *in vivo* pyroptosis and the accompanied products that directly kill bacteria during microbial infection.

In seeking for the mechanism underlying berberine's beneficial effects on diabetes and insulin-resistance, it has been found that berberine is an agonist of AMP-activated protein kinase (AMPK) [26, 27]. AMPK is a master regulator for cellular energy homeostasis, being activated under stresses that deplete cellular ATP supplies such as low glucose, hypoxia, ischemia, inflammation and heat shock [37]. In addition, multiple cellular activities other than energy homeostasis can be regulated by AMPK signaling, including cell proliferation, polarization, protein translation and lipid metabolism [38, 39]. Activation of AMPK by berberine may improve cellular glucose and lipid metabolism in patients with diabetes [26], although this remains debatable [40]. Previous studies have indicated that AMPK activation can enhance the phagocytic and bacterial killing abilities of neutrophils and macrophages [30, 31, 41]. This suggested that activation of AMPK by berberine might have correlated with its effect on enhancing inflammasome activation, pyroptosis and release of IL-1β. Two lines of evidence in this study have supported this hypothesis. First, ATP triggering induced AMPK activation while berberine simultaneously augmented AMPK signaling and inflammasome activation, as well as pyroptosis and IL-1β release, upon ATP treatment. Second, AMPK signaling blockade by specific inhibitor compound C or *AMPKα* knockdown markedly attenuated ATP-induced pyroptosis and reversed the augmentation of pyroptosis by berberine. Our results highlight a potential link between berberine-mediated AMPK signaling and augmentation of ATP-induced NLRP3 inflammasome activation, which deserves further investigation to elucidate the precise mechanism underlying this process.

In conclusion, we found that berberine could markedly augment ATP-induced inflammasome activation, as well as pyroptosis and IL-1β release, in macrophages. This activity of berberine might be attributed to enhancing AMPK signaling. More importantly, berberine-mediated augmentation of inflammasome activation increased the bactericidal ability of macrophages and/or neutrophils *in vitro* and *in vivo*, leading to increased survival of mice upon bacterial infection. These findings highlight an indirect antimicrobial effect of berberine against bacterial infection by enhancing the functions of macrophages and probably other immune cells.

## MATERIALS AND METHODS

### Reagents and antibodies

Berberine (B3251), adenosine triphosphate (ATP) (A6419), dimethyl sulfoxide (DMSO), propidium iodide (PI), Hoechst 33342, Tween-80, lipopolysaccharide (LPS) (*Escherichia coli* O111:B4) (L4391), and compound C (C.C) were purchased from Sigma-Aldrich (St. Louis, MO, USA). Berberine was dissolved in DMSO at 50 mM, stored at −20°C. ATP was dissolved in phosphate-buffered saline (PBS) and stored at −20°C. Thioglycollate medium (Brewer modified) (TG) was obtained from Becton Dickinson (Sparks, MD, USA). Opti-MEM, Dulbecco's Modified Eagle's Medium (DMEM) medium with high glucose, fetal bovine serum (FBS), streptomycin and penicillin were products of ThermoFisher/Gibco (Carlsbad, CA, USA). Antibodies against caspase-1p10 (sc-514) and actin (sc-1616-R) were obtained from Santa Cruz (Dallas, TX, USA). The antibody to NLRP3 (AG-20B-0014) was purchased from Adipogen AG (Liestal, Switzerland). Specific antibodies against IL-1β (#12242), HMGB1 (#3935), AMPKα (#5832), phospho-AMPKα (Thr172) (#2535), ASC (#67824), β-tubulin (#2128) and horse-radish peroxidase (HRP)-conjugated goat anti-mouse/rabbit IgG were bought from Cell Signaling Technology (Danvers, MA, USA). CF568 goat-anti-rabbit IgG (H+L), highly cross-adsorbed (#20103-1) was obtained from Biotium (Hayward, CA, USA). Anti-mouse CD11b FITC (#11-0112) and anti-mouse F4/80 PE (12-4801) were obtained from eBioscience (San Diego, CA, USA). Anti-mouse Ly-6G (Gr-1) PerCP (#127653) was bought from BioLegend (San Diego, CA, USA).

### Experimental animals

Female C57BL/6 mice (6-8 weeks of age) were purchased from the Experimental Animal Center of Southern Medical University (Guangzhou, China). All animal experiments were performed in accordance with the guidelines for the care and use of animals approved by the Committee on the Ethics of Animal Experiments of Jinan University.

### Cell culture

The murine macrophages cell line J774A.1 was obtained from the Kunming Cell Bank of Type Culture Collection Chinese Academy of Sciences (Kunming, China). Cells were maintained in DMEM supplemented with 10% FBS, 100 U/ml penicillin, 100 μg/ml streptomycin, and 2 mM L-glutamine (complete DMEM medium) at 37°C in a humidified incubator of 5% CO_2_ and sub-cultured every 2-3 d by using a cell scraper to detach cells.

### Isolation of TG-elicited peritoneal macrophages

The separation of TG-elicited peritoneal macrophages (TGPMs) was performed as described previously [42]. Briefly, mice were administered intraperitoneally with 1 ml of 3% TG medium and sacrificed 4 d later. Peritoneal macrophages were isolated with 2 ml washing buffer (germ-free PBS containing 0.5 mM EDTA and 5% calf serum). The cells were collected by centrifugation at 300 ×g for 10 min, re-suspended in complete DMEM medium and seeded in plates. After 2-h incubation at 37°C in a humidified incubator of 5% CO_2_, the unattached cells were removed and the adherent macrophages were used for experiments. The purity of TGPMs was assessed by flow cytometry using FITC-labeled anti-mouse CD11b and PE-labeled anti-mouse F4/80 antibodies and was routinely > 95% (Supplementary Figure S7A). Their morphological characteristics are shown in Supplementary Figure S7B.

### Bone marrow-derived macrophage culture

Mouse bone marrow-derived macrophages (BMDMs) were differentiated as reported previously [15]. In brief, mice were sacrificed and the bone marrow in hind femora and tibias was flushed out with 10 ml of sterile PBS and collected by centrifugation at 300 ×g for 5 min at 4°C. The bone marrow cells were re-suspended in BM-Mac medium (DMEM supplemented with 10% FBS and 20% M-CSF-conditioned medium from L929 cells) and 5×10^6^ cells in 10 ml BM-Mac medium were cultured in a 10-cm petri dish at 37°C in a humidified incubator of 5% CO_2_. Each petri dish was replenished with 5 ml BM-Mac medium after 2 days. The culture medium was removed and replaced with 10 ml of fresh BM-Mac medium after 4 days. BMDM cells were ready for experiments after 6 days. The cells were collected with a cell-scraper and cultured in complete DMEM medium overnight in 24-well plates at 1.5×10^5^ cells/well (0.5 ml) or in 6-well plates at 1.5×10^6^ cells/well (2.0 ml). The BMDM purity was assessed by flow cytometry using FITC-labeled anti-mouse CD11b and PE-labeled anti-mouse F4/80 antibodies and was routinely > 95% (Supplementary Figure S7A). Their morphological characteristics are shown in Supplementary Figure S7B.

### Flow cytometry

For phenotyping analysis, peritoneal exudate cells were isolated and washed with PBS-F (PBS containing 0.1% NaN_3_ and 3% calf serum), followed by staining with FITC labeled anti-mouse CD11b in conjunction with PerCP labeled anti-mouse Ly-6G (Gr-1) at 4°C for 30 min. After washing with PBS-F, cells were fixed with 4% paraformaldehyde in PBS and then analyzed on a flow cytometer (FACSCalibur; Becton Dickinson). Data were acquired and analyzed by using the CELLQuest software (Becton Dickinson).

### Cell death assay

Cell death was measured by PI incorporation as described previously [25, 42]. Cells were seeded in 24-well plates and primed with 500 ng/ml LPS for 4 h. Subsequently, the cells were treated with various concentrations of berberine for 1 h followed by appropriate concentration of ATP for indicated time periods in Opti-MEM. PI (2 μg/ml) solution containing Hoechst 33342 (5 μg/ml) was added to cell culture media at room temperature for 10 min, and cells were observed immediately by live imaging using Zeiss Axio Observer D1 microscope equipped with a Zeiss LD Plan-Neofluar 20×/0.4 Korr M27 objective lens (Carl Zeiss MicroImaging GmbH, Göttingen, Germany). Fluorescence images were captured with a Zeiss AxioCam MR R3 cooled CCD camera controlled with ZEN software (Carl Zeiss).

### Small interfering RNA (siRNA)

The siRNA (5′-GCA GAA GUG UGU AGA GCA A-3′) duplexes targeting mouse *AMPKα1* and negative control (NC) siRNA were designed and synthesized by RiboBio (Guangzhou, China). siRNA transfection was performed using Lipofectamine RNAiMAX (ThermoFisher) according to the instruction manual. The siRNA was added to each well at a final concentration of 100 nM. Cells were cultured in DMEM medium containing 10% FBS for 72 h.

### Precipitation of soluble proteins

Soluble protein secreted into culture supernatants (equal volume for each sample) were precipitated with 0.15% sodium deoxycholate plus 7.2% trichloroacetic acid as previously described [43]. The precipitated proteins were re-dissolved in equal volume of 1× sodium dodecylsulfate-polyacrylamide gel electrophoresis (SDS-PAGE) sample loading buffer and subjected to western blot analysis for released HMGB1, mature IL-1β and caspase-1p10.

### Western blot analysis

Western blotting was performed essentially as previously described [44]. Total proteins were separated by SDS-PAGE followed by electrotransfer to polyvinylidene difluoride (PVDF) membranes (Hybond-P; GE Healthcare Life Sciences, Piscataway, NJ, USA). The membranes were blocked and incubated with primary antibody (1:1000), followed by incubation with appropriate HRP-conjugated secondary antibody (goat anti-mouse/rabbit/rat IgG). Bands were revealed with an enhanced chemluminescence kit (BeyoECL Plus; Beyotime, Haimen, China) and recorded by X-ray films (Carestream, Xiamen, China). The blot images were captured by FluorChem8000 imaging system (AlphaInnotech, San Leandro, CA, USA).

### Immunofluorescence microscopy

Immunofluorescence analysis was performed as previously described [45]. Briefly, BMDMs were seeded in glass-bottomed dishes (4×10^5^ cells/dish) and cultured at 37°C overnight. Cells were primed with 500 ng/ml LPS for 4 h. Then the cells were treated with berberine or vehicle for 1 h followed by treatment with 2 mM ATP for 30 min in Opti-MEM. After these treatments, the cells were fixed in 4% paraformaldehyde for 15 min, and permeabilized with 2 ml cold methanol (−20°C) for 10 min. Then the cells were incubated with ASC antibody (1:300) overnight, followed by staining with CF568-conjugated goat-anti-rabbit IgG for 1 h. After Hoechst 33342 solution (5 μg/ml in PBS) was added to stain the nuclei for 10 min, the cells were observed under a Zeiss Axio Observer D1 microscope with a Zeiss LD Plan-Neofluar 40×/0.6 Korr M27 objective (Carl Zeiss MicroImaging GmbH, Göttingen, Germany). Fluorescence images were captured by a Zeiss AxioCam MR R3 cooled CCD camera controlled with ZEN software (Carl Zeiss).

### Bacterial infection

Bacterial infection was performed as previously described [19, 45]. Briefly, *E. coli* strain DH5α was grown in Luria Broth (LB) media and bacterial cell density was determined using an ultraviolet-visible spectrophotometer (NanoDrop2000, Thermo Scientific) and the corresponding colony-forming units (CFUs) were determined on LB agar plates. Mice were acclimated for one week and intragastrically administered with berberine solution or vehicle (2% Tween-80 in PBS) once a day for 3 consecutive days. Viable *E. coli* cells (2×10^9^ CFU/mouse) in 0.5 ml of PBS were injected into the peritoneal cavity (i.p.). Mouse survival was monitored every 6 h for 4 days. In a separate experiment, mice were sacrificed at indicated time points and peritoneal lavage fluids were collected with 1.5 ml PBS. Serial dilutions of the lavage fluids were incubated overnight at 37°C on LB agar plates; CFUs of bacteria were counted. The colon tissues were collected for western blotting and histopathological analysis, respectively.

### Detection of soluble cytokines

Soluble IL-1β in mouse peritoneal lavage fluids and in culture supernatants of macrophages was determined by Cytometric Bead Array (CBA) Mouse IL-1β Flex Set (BD Biosciences, San Jose, CA, USA) according to the manufacturer's instructions. Data were acquired using CELLQuest software on a flow cytometer (FACSCalibur; Becton Dickinson).

### Histopathology

After mice were sacrificed, the liver and colon were fixed in 4% neutral formaldehyde, and stained with hematoxylin and eosin (H&E). Images were captured by the Zeiss Axio Observer D1 microscope armed with a color CCD (Zeiss Axio Observer D1).

### Statistical analysis

All experiments were performed three times independently, with one representative experiment shown. Data were expressed as mean ± standard deviation (SD). Statistical analysis was performed using GraphPad Prism 5.0 (GraphPad Software Inc, San Diego, CA, USA). One-way analysis of variance (ANOVA) followed by Tukey post-hoc test and unpaired Student's *t-*test were used to analyze the statistical significance among multiple groups and between two groups, respectively. Kaplan-Meier survival curves were adopted for analysis of mouse survival, and the statistical difference between 2 groups was determined using the log-rank (Mantel-Cox) test. *P*-values < 0.05 were considered statistically significant.

## SUPPLEMENTARY MATERIAL


