# Supplementary material for: Berberine augments ATP-induced inflammasome activation in macrophages by enhancing AMPK signaling
Source: Oncotarget. 2016 Dec 12;8(1):95–109. doi: 10.18632/oncotarget.13921 (PMC5352208; doi:10.18632/oncotarget.13921)
Supplement: Supplementary file 1 [file oncotarget-08-95-s001.pdf]

Berberine augments ATP-induced inflammasome activation in macrophages by enhancing AMPK signaling

Supplementary Material

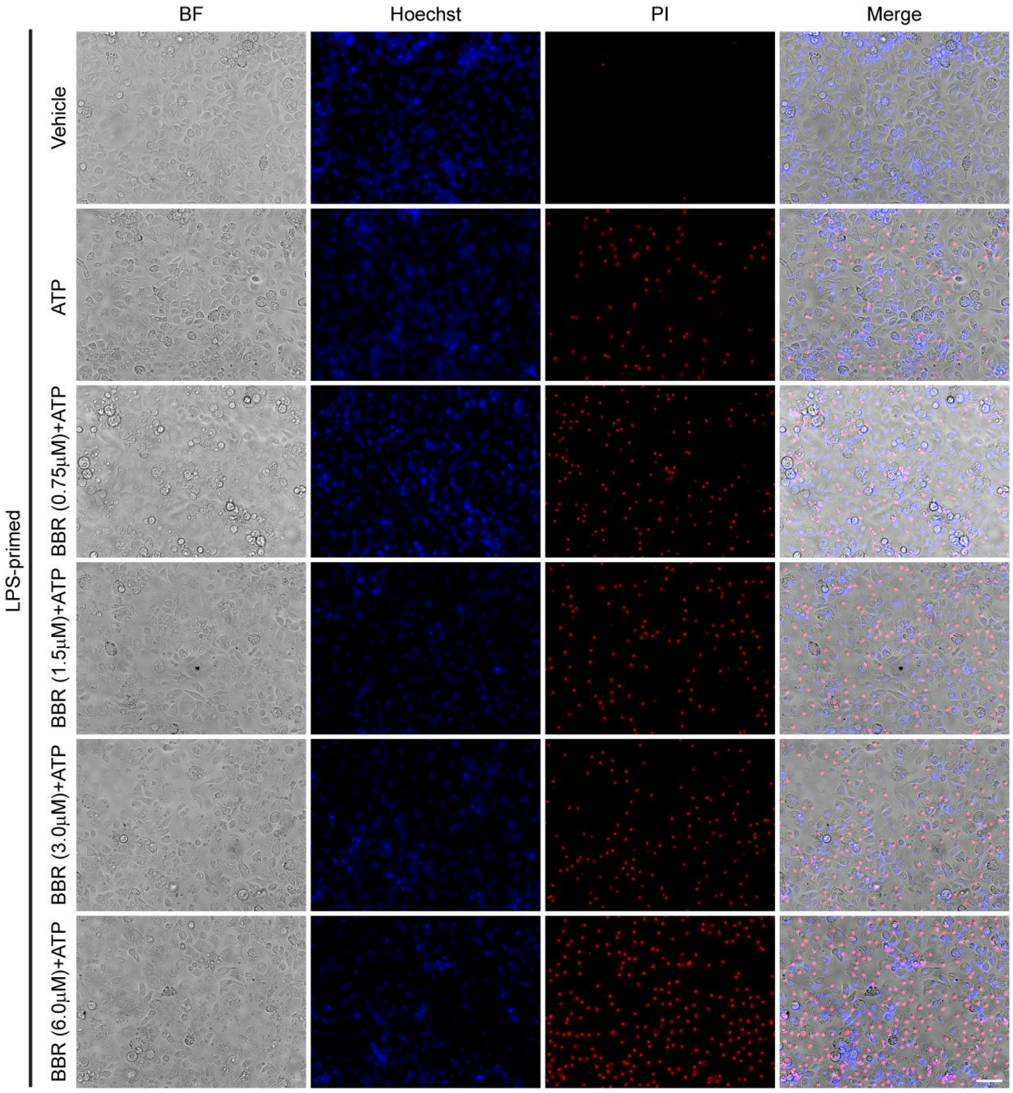

Supplementary Figure S1: Morphological characteristics of cell death induced by co-treatment with ATP and berberine in TG-elicited peritoneal macrophages. Cells were stimulated with LPS (500 ng/ml) for 4 h and pre-treated with various concentrations of berberine for 1 h followed by co-treatment with ATP (2 mM) for 30 min in the absence of LPS. Cell death was assayed by propidium iodide (PI) (red) and Hoechst 33342 (blue) staining and fluorescent images were captured by fluorescence microscopy, merged with bright-field images. One set of representative images of three independent experiments are shown. Scale bar, 50 μm; BF, bright field; BBR, berberine.

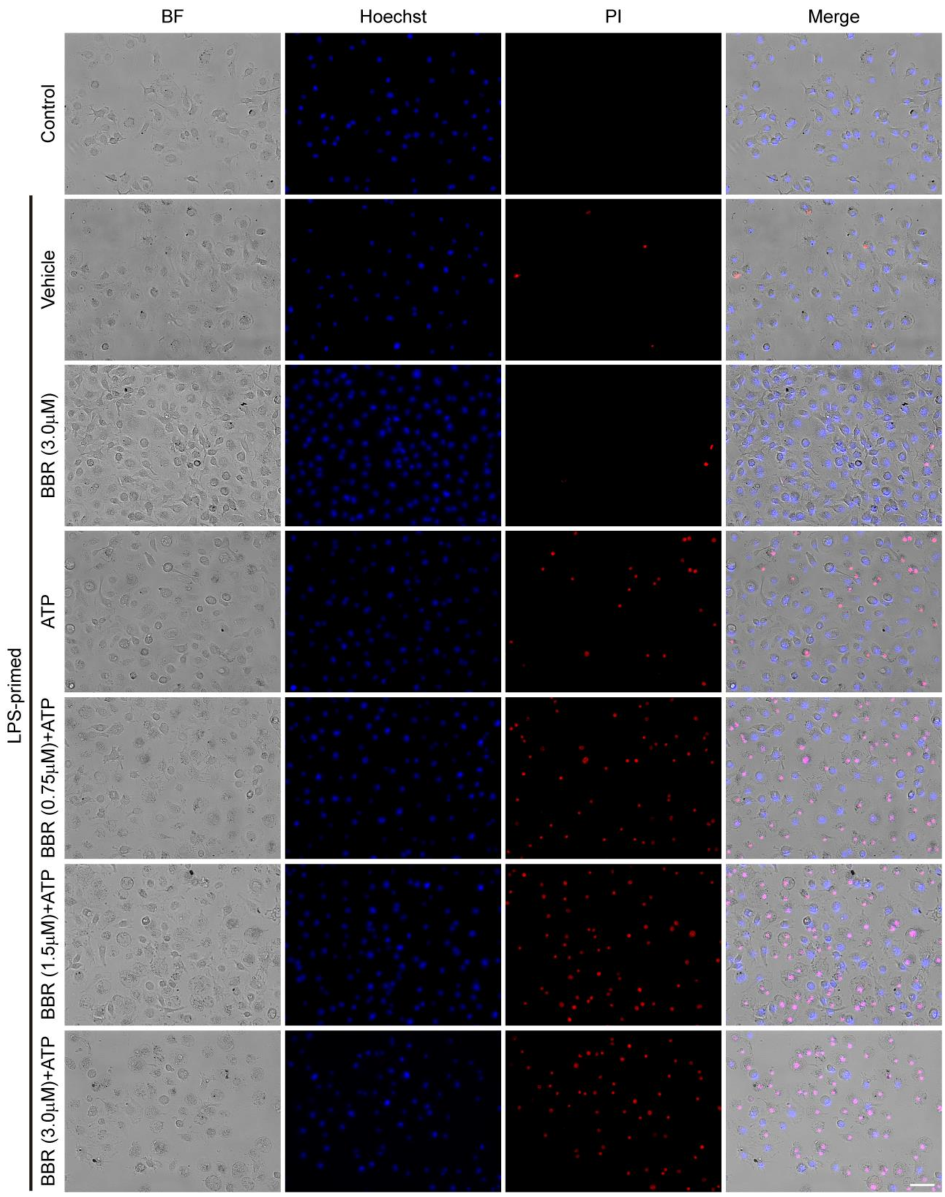

Supplementary Figure S2: Morphological characteristics of cell death induced by co-treatment with ATP and berberine in bone marrow-derived macrophages. Cells were stimulated with LPS (500 ng/ml) for 4 h and pre-treated with graded doses of berberine for 1 h followed by co-treatment with ATP (2 mM) for additional 30 min in the absence of LPS. Cell death was assayed by propidium iodide (PI) (red) and Hoechst 33342 (blue) staining and fluorescent images were captured by fluorescence microscopy, merged with bright-field images. One set of representative images of three independent experiments are shown. Scale bar, 50 μm; BF, bright field; BBR, berberine.

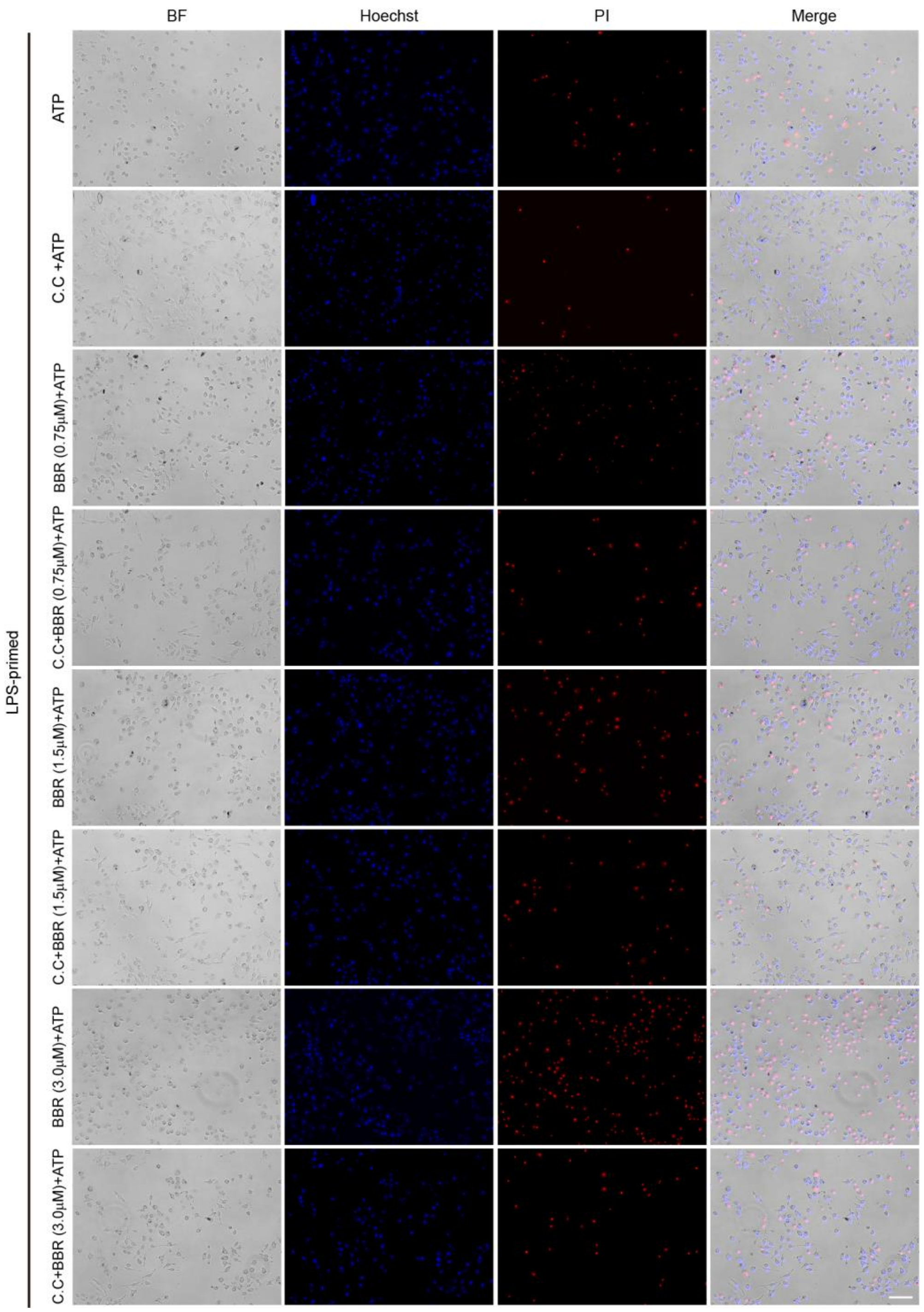

Supplementary Figure S3: Morphological characteristics of cell death induced by co-treatment with ATP and berberine in J774A.1 cells in the presence of AMPK inhibitor compound C (C.C). Cells were stimulated with LPS (500 ng/ml) for 4 h and pre-treated with C.C (20 μM) for 1 h before incubation with various concentrations of berberine for 1 h followed by co-treatment with ATP (3 mM) for additional 1 h. Cell death was assayed by propidium iodide (PI) (red) and Hoechst 33342 (blue) staining and fluorescent images were captured by fluorescence microscopy, merged with bright-field images. One set of representative images of three independent experiments are shown. Scale bar, 100 μm; BF, bright field; BBR, berberine.

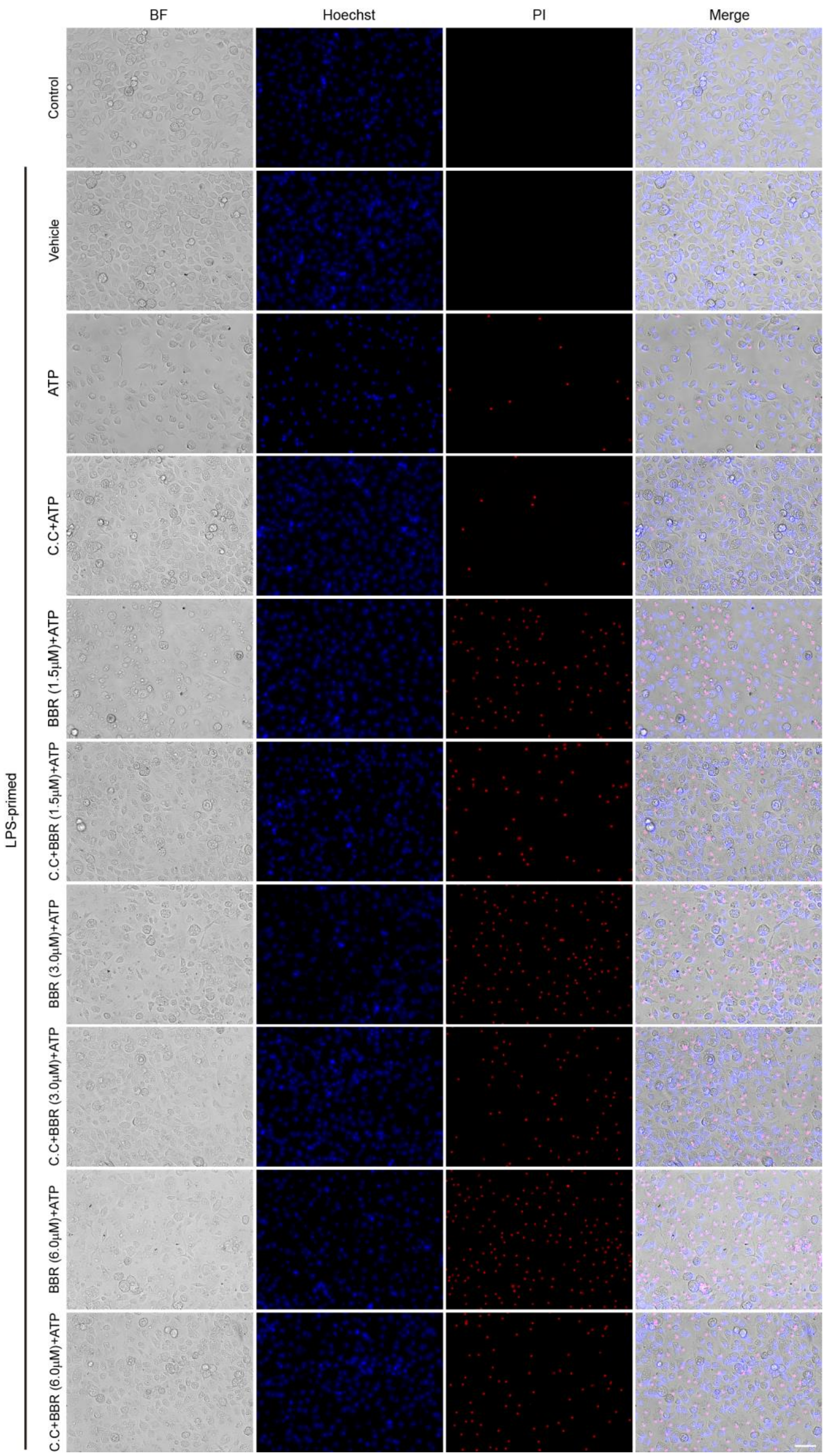

Supplementary Figure S4: Morphological characteristics of cell death induced by

co-treatment with ATP and berberine in TG-elicited peritoneal macrophages in the presence of AMPK inhibitor compound C (C.C). Cells were stimulated with LPS (500 ng/ml) for 4 h and then pre-treated with C.C (20 μM) for 1 h before incubation with various concentrations of berberine for 1 h and co-treatment with ATP (2 mM) for additional 30 min. Cell death was assayed by propidium iodide (PI) (red) and Hoechst 33342 (blue) staining and fluorescent images were captured by fluorescence microscopy, merged with bright-field images. One set of representative images of three independent experiments are shown. Scale bar, 50 μm; BF, bright field; BBR, berberine.

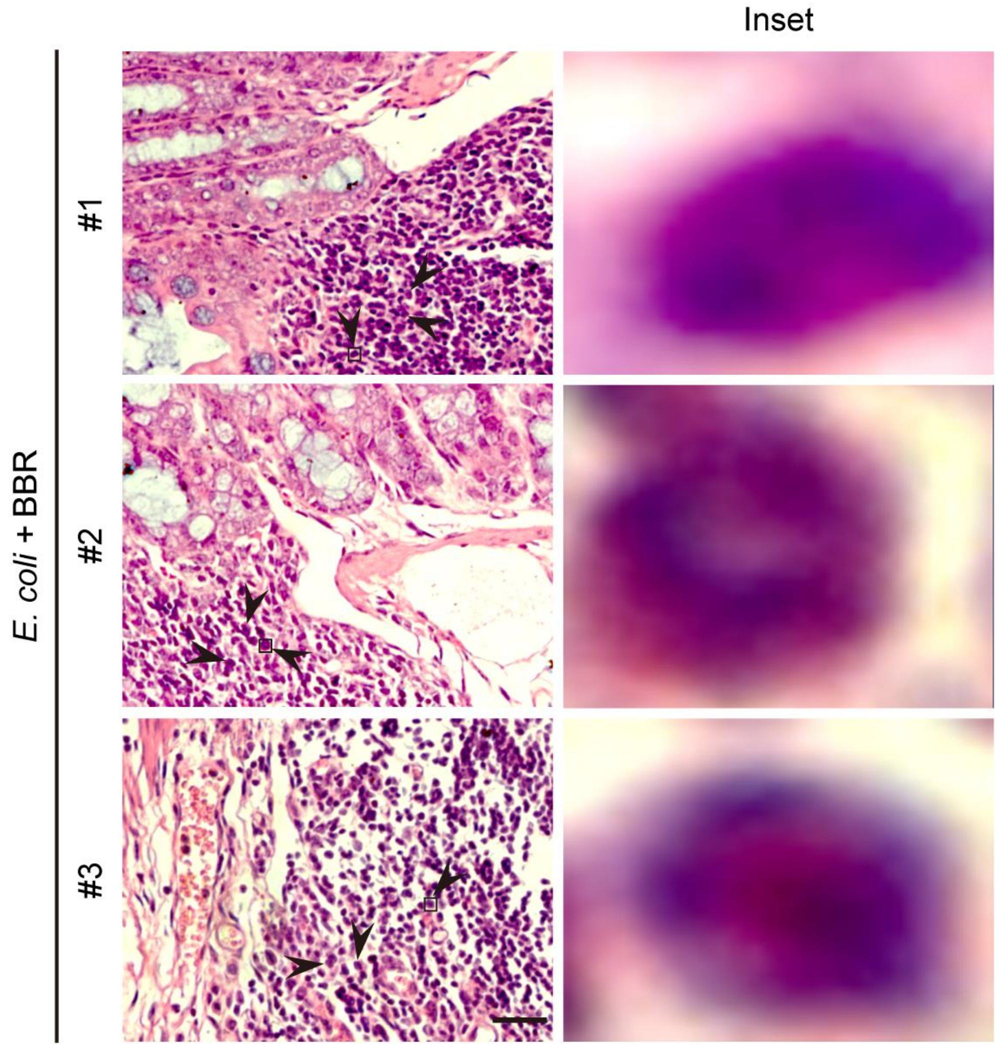

Supplementary Figure S5: Berberine increased inflammatory cell recruitment in the colon. Mice were administered (i.g.) with berberine (100 mg/kg body weight) once a day for 3 consecutive days prior to injection (i.p.) with viable *E. coli* (2×10^9 CFU/mouse). 8 h after infection, mice were sacrificed and the colon was fixed in 4% neutral formaldehyde, and stained with hematoxylin and eosin (H&E). Representative images from each mouse are shown. Arrow heads indicate infiltrated polymorphonuclear cells (neutrophils) in the colon tissues. Nuclear morphology was shown in enlarged insets. Scale bar, 20 μm; BBR, berberine.

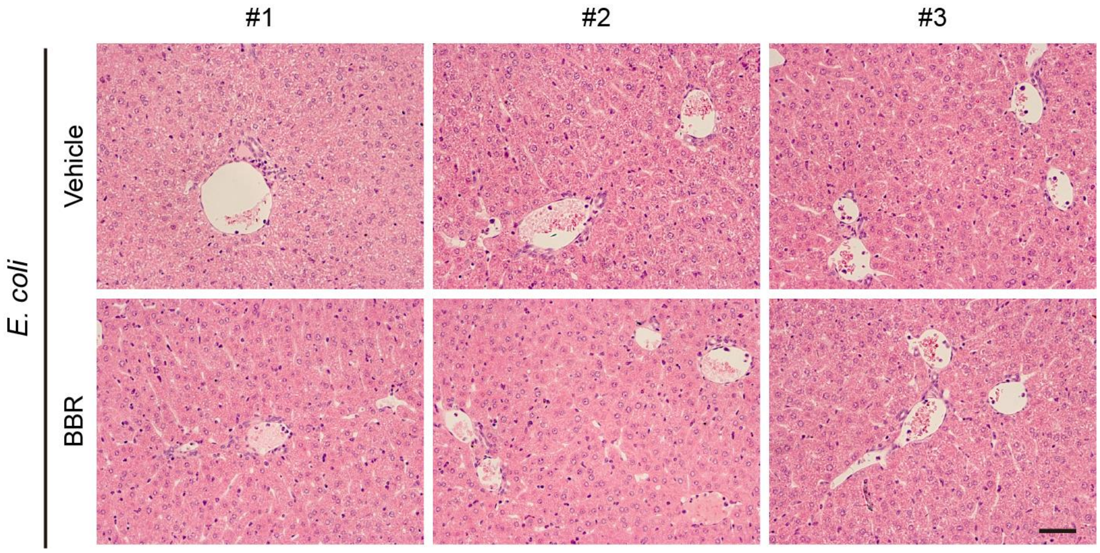

Supplementary Figure S6: Berberine did not affect the infiltration of inflammatory cells in the liver. Mice were administered (i.g.) with berberine (100 mg/kg body weight) or vehicle (2% Tween-80 in PBS) once a day for 3 consecutive days prior to injection (i.p.) with viable *E. coli* (2×10^9 CFU/mouse). 8 h after infection, mice were sacrificed and the liver was fixed in 4% neutral formaldehyde, and stained with hematoxylin and eosin (H&E). Representative images from each mouse are shown. Scale bar, 50 μm; BBR, berberine.

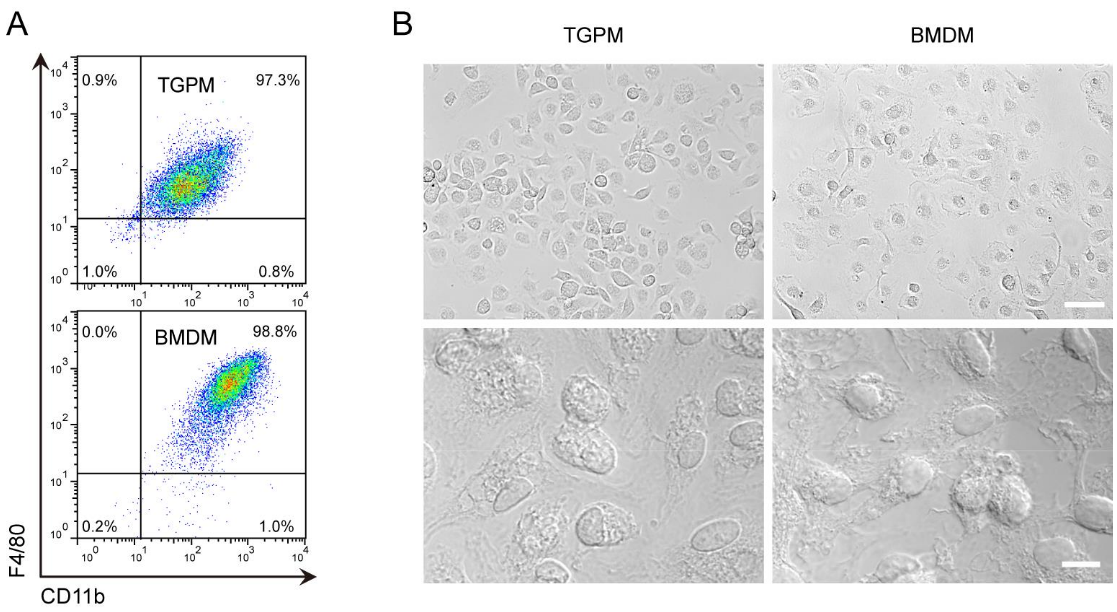

Supplementary Figure S7: The purity and morphological characteristics of TG-elicited peritoneal macrophages (TGPMs) and bone marrow-derived macrophages (BMDMs). A. The purity of TGPMs and BMDMs were analyzed by flow cytometry using FITC-labeled anti-mouse CD11b and PE-labeled anti-mouse F4/80 antibodies. Representative dot-plots from one of three independent experiments are shown. **B.** Bright-field images of TGPMs and BMDMs were captured by using the Zeiss Axio Observer D1 microscope. Scale bars, 50 μm (upper panel) and 10 μm (bottom panel).
